# Defective Protein Catabolism in Atherosclerotic Vascular Inflammation

**DOI:** 10.3389/fcvm.2017.00079

**Published:** 2017-12-07

**Authors:** Takuro Miyazaki, Akira Miyazaki

**Affiliations:** ^1^Department of Biochemistry, School of Medicine, Showa University, Tokyo, Japan

**Keywords:** calpain, autophagy, ubiquitin proteasome, nitric oxide, inflammasome, mechanotransduction, apoptosis, efferocytosis

## Abstract

Vascular inflammation in atheroprone vessels propagates throughout the arterial tree in dyslipidemic patients, thereby accelerating atherosclerotic progression. To elucidate the mechanism of vascular inflammation, most previous studies have focused on inflammation-related signals that are sent in response to vasoactive stimuli. However, it is also important to understand how normal blood vessels become defective and start degenerating. Growing evidence suggests that major protein catabolism pathways, including the ubiquitin-proteasome, autophagy, and calpain systems, are disturbed in atheroprone vessels and contribute to the pathogenesis of atherosclerosis. Indeed, dysregulation of ubiquitin–proteasome pathways results in the accumulation of defective proteins in blood vessels, leading to vascular endothelial dysfunction and apoptosis in affected cells. Impaired autophagy-lysosomal degradation affects smooth muscle cell transformation and proliferation, as well as endothelial integrity and phagocytic clearance of cellular corpses. Dysregulation of the calpain system confers proatherogenic properties to endothelial cells, smooth muscle cells, and macrophages. In this review article, we will discuss the current information available on defective protein catabolism in atheroprone vessels and its potential interrelation with inflammation-related signals.

## Introduction

Atherosclerosis is a chronic inflammatory disease accompanied by the intimal thickening of systemic arterial walls (1, 2). Rupture of vulnerable plaques as well as thrombotic/embolic occlusion and arterial narrowing can be causal of lethal ischemic disease, including acute coronary syndromes/myocardial infarction (3). Regarding the pathogenic cues that contribute to atherosclerosis, the majority of previous atherosclerosis studies support the hypothesis that atherosclerosis is driven through reactive oxygen species (ROS)-mediated oxidative stress, which leads to the induction of numerous inflammatory elements, such as adhesion molecules in vascular endothelial cells (ECs) and proinflammatory cytokines *via* redox-sensitive transcription factors (2). During prolonged vascular inflammation, degenerative insults, such as increased numbers of apoptotic cells, remodeling of the extracellular matrix (ECM), breakdown of elastic lamella, and the dysfunction of ECs, emerge in atheroprone vessels and accelerate atherosclerosis-related complications (2). Although earlier clinical trials investigated the efficacy of antioxidants, these agents failed to reverse cardiovascular death (4); in contrast, they were effective in ameliorating acute inflammation, including the acute phase of stroke (5). Therefore, in addition to the mechanisms underlying acute inflammatory insults, it is important to understand how blood vessels shift toward degenerative status in chronic vascular disease. In this regard, accumulating evidence indicates that defects in protein catabolism systems, which consist of a variety of intracellular proteases, critically contribute to inflammatory vascular degeneration. Autophagy, an essential intracellular process mediated by the lysosomal degradation of cytoplasmic components, is detectable in every tissue and mediates the nutrient turnover, particularly in cells under starving conditions. Although this system is involved in sustaining cellular metabolism, homeostasis, and survival (6), autophagic flux was detected in atheroprone vessels (7). Furthermore, the ubiquitin–proteasome system, which acts as a threshold machinery for protein catabolism (8), is regulated through proinflammatory cytokines such as interferon-γ (9), oxidized low-density lipoprotein (LDL) (10), and oxidized cholesterol (11). In addition to their roles in protein degradation, several classes of proteases transduce cellular signaling through their ability to process target proteins in stress-inducing environments. Indeed, calpain, a Ca^2+^-dependent intracellular cysteine protease, is activated in response to several classes of cytokines, growth factors, lysophospholipids, and physical stresses (12), thereby participating in degenerative vascular disorders (13, 14). In this review, we summarize the recent achievements in the proinflammatory and proatherogenic defects of these protein catabolism pathways. In addition, we will discuss anti-atherosclerosis strategies that target defective protein catabolism.

## Defective Protein Catabolism Underlies Atherosclerosis

### Autophagy

Autophagy is a self-degenerative process that participates in organelle turnover, and the recycling of cytoplasmic components as well as protein degradation in response to extracellular stresses. Currently, autophagy can be categorized into three classes (6). Microautophagy is a non-selective lysosomal process describing the direct engulfment of cytoplasmic cargos by lysosomes. This process is accomplished by the inward invagination of cargos into the lysosomal membrane. In contrast, chaperone-mediated autophagy enables the selective degradation of cytoplasmic proteins by recognizing chaperone proteins. During this process, lysosomal-associated membrane protein type 2A on the lysosomal membrane recognizes the target proteins *via* their chaperone (e.g., heat shock cognate protein of 70 kDa), allowing internalization of the target protein into lysosomes. Macroautophagy is a process whereby cytoplasmic components are degraded by lysosomes, and it is accompanied by the formation of autophagosomes. These autophagosomes are cytosolic double-layered membrane vesicles, in which cytoplasmic components are separated from the cytoplasmic environment, and which finally fuse with a lysosome where lysosomal digestion occurs. One of the key regulators of autophagy is the kinase mammalian target of rapamycin (mTOR), which negatively regulates autophagy (6). Autophagosome formation is mediated by *Atg* genes, which is regulated by Atg12–Atg5–Atg16L1 and LC3–PE (*Atg8* homolog) complexes (6). It was reported that nutrient supply by macroautophagic protein catabolism sustained anabolic reactions to generate macromolecules, such as nucleic acids, proteins, and organelles. As a result, the nutrient supply can exert a prosurvival mechanism, particularly under starving conditions (15). In contrast, the overactivation of autophagic systems elicits cell death, which may be mediated by a process morphologically distinct from apoptosis (16). The contribution of macroautophagy to tumorigenesis, infection, diabetes, and cardiovascular diseases was described previously (6, 7, 15). In vascular systems, macroautophagy is detectable in macrophages in atherosclerotic lesions as well as in vascular-resident cells, including ECs and vascular smooth muscle cells (VSMCs), where it participates in physiologic and pathogenic responses (6).

Razani et al. reported that macroautophagy was associated with the pathogenesis of atherosclerosis (17). The expression of p62 protein, but not of p62 mRNA, was potentiated in lesional macrophages suggesting insufficient autophagy in these cells. Macrophage-specific deficiency of *Atg5* accelerated the development of atherosclerosis in *Apoe^−/−^* mice by insufficient autophagy in cells. Furthermore, Liao et al. also reported abnormal expression patterns of LC3 and the presence of p62-positive cells in atheromas in atherogenic mice, indicating impaired autophagy in the lesions (18). Accordingly, macrophage-specific *Atg5* deficiency augmented the expansion of lesional necrotic cores and progression of atherosclerosis. Therefore, insufficient autophagy might decrease the stability of atherosclerotic plaques as well as its pathogenic implications.

### Ubiquitin–Proteasome System

The ubiquitin proteasomal systems are part of the central protein catabolism machinery in cells and are involved in the degradation of misfolded proteins, as well as normal protein degradation. These systems are also implicated in cellular events, including antigen presentation in immune cells (19) and the cell cycle (20). The polyubiquitination of target molecules triggers proteasomal degradation, which is driven by E1 ubiquitin-activating enzymes, E2 ubiquitin-conjugating enzymes, and E3 ubiquitin-protein ligases (21). Following the ubiquitination process, ubiquitinated target proteins are recognized by proteasomal complexes and are proteolytically degraded into peptides. In this process, the 26S proteasome comprising regulatory particle 19S and core particle 20S, has a crucial role in the proteasomal systems (9). Similar to autophagic systems, dysfunctional proteasomes can cause degenerative diseases, such as Alzheimer’s disease (22), Parkinson’s disease (22), Huntington’s disease (22), and atherosclerosis (23, 24). An atherosclerosis study in high fat diet-fed pigs identified that proteasomal activity was elevated in coronary arteries during the progression of atherosclerosis (25). However, in a human atherosclerosis study, the carotid atherosclerotic lesions in subjects complicated with transient ischemic attack or stroke exhibited lower proteasomal activity compared with that in asymptomatic subjects (26). These studies suggest that proteasomal activity declines in advanced atherosclerotic plaques, whereas it is sustained in mild lesions. In addition to these observations, animal experiments with pharmacological proteasomal intervention have been performed. Herrmann et al. examined the long-term administration of the proteasome inhibitor MLN-273 in high cholesterol diet-fed pigs and identified the elevation of oxidative stress and exacerbation of atherosclerosis (25). Conversely, Wilck et al. reported that a moderate dose of bortezomib (also known as PS-341) led to partial proteasomal inhibition in high cholesterol diet-fed *Ldlr^−/−^* mice, which reduced oxidative stress and NADPH oxidase 4 expression levels in the aortae, thereby suppressing atherosclerosis (27). Therefore, it hardly defines pathophysiologic roles of proteasomal systems in atherosclerosis. It is well known that macrophage cytotoxicity is prominent in advanced atheromas, while vascular inflammation is sustained throughout moderate-to-advanced atherosclerotic lesions (1). Thus, it is suspected that the contribution of proteasomal systems to the pathogenesis of atherosclerosis is substantially dependent upon the stage of the disease.

### Calpain Systems

The calpain family comprises Ca^2+^-dependent intracellular proteases (28–30). These proteolytic systems are unique because calpains modulate their substrates through limited proteolytic cleavage in addition to proteolytic degradation. The calpain system comprises the endogenous inhibitor calpastatin as well as 15 homologs of the catalytic subunits, and 2 regulatory subunit homologs in mammals (28–30). Calpain catalytic subunits are categorized into conventional and unconventional subtypes (28–30). The conventional subtype consists of two isozymes, calpain-1 and calpain-2, which are ubiquitously expressed in vertebrates (28–30). These are heterodimers of the catalytic subunits, CAPN1 and CAPN2, which form calpain-1 and calpain-2 isozymes, respectively, together with their common regulatory subunit, CAPNS1 (28–30). The substrate selectivity of those conventional calpains is currently unclear, but they exhibit enzymatically distinct properties. Indeed, calpain-1 and calpain-2 require micromolar and millimolar levels of Ca^2+^ for half-maximal activation, respectively. It was reported that a variety of biophysical and biochemical stimuli elevate intracellular Ca^2+^ levels (31–33); therefore, the activity of these protease systems has been the focus of numerous pathophysiologic studies (13, 14). Our previous investigation indicated that oxidized or enzymatically modified LDL or its component, lysophosphatidylcholine, induced calpain-2; accordingly, this molecule is enriched in ECs in human and mouse atheromas (34). Calpain-2 exerts pro-atherogenic roles because the calpain inhibitors calpeptin and ALLM counteracted aortic atherosclerotic development in high cholesterol diet-fed *Ldlr^−/−^* mice. It was also reported that the calpain inhibitor BDA-410 inhibited atherosclerotic development in angiotensin II-infused *Ldlr^−/−^* mice (35). Furthermore, angiotensin II- or hypercholesterolemia-induced atherosclerosis in *Ldlr^−/−^* mice was suppressed by the transgenic overexpression of calpastatin; in contrast, abdominal aneurysmal formation, a complication of angiotensin II-infused mice, was unaltered (36). A study using angiotensin II-infused mice showed that calpain-1 in myeloids and calpain-2 in leukocytes were responsible for atherogenesis (36). Collectively, conventional calpains participate in pathophysiologic regulation in ECs and leukocytes, thereby facilitating atherosclerosis.

Our previous data identified that unconventional calpains also contribute to atherogenesis (37). Expression analysis in *Ldlr^−/−^* mice showed that *Capn2, Capn6*, and *Capn9* were upregulated in atheroprone aortae. *Capn6* deficiency decelerated the progression of atherosclerosis in *Ldlr^−/−^* mice, while *Capn9* deficiency did not have any effect. Calpain-6 is a non-proteolytic calpain because the cysteine residue in its CysPc domain is substituted with a lysine (28–30). This molecule is expressed preferentially in macrophages in advanced atheromas in humans and mice (37). Interestingly, calpain-6 was originally identified in the skeletal muscle, cartilage, and heart of a murine fetus (38); however, its expression is absent in adult tissues with the exception of the placenta under non-disease conditions (39). Therefore, calpain-6 might be potentiated under developmental and pathogenic conditions. Regarding atherosclerosis, it is likely that calpain-6 is upregulated in macrophages after cells infiltrate into lesions, whereas it is absent in bone marrow cells (37). Furthermore, calpain-6 induction can be reproduced in cultured bone marrow-derived macrophages by cytokine stimulation. In particular, tumor necrosis factor-α markedly induced calpain-6. Atherogenesis appears to be dependent upon myeloid *Capn6* but not upon other cell lineages in mice. Therefore, the proatherogenic transformation of macrophages by calpain-6 is responsible for atherosclerotic disease.

## Contribution of Defective Protein Catabolism to Atherogenic Inflammation

**Figure 1:**
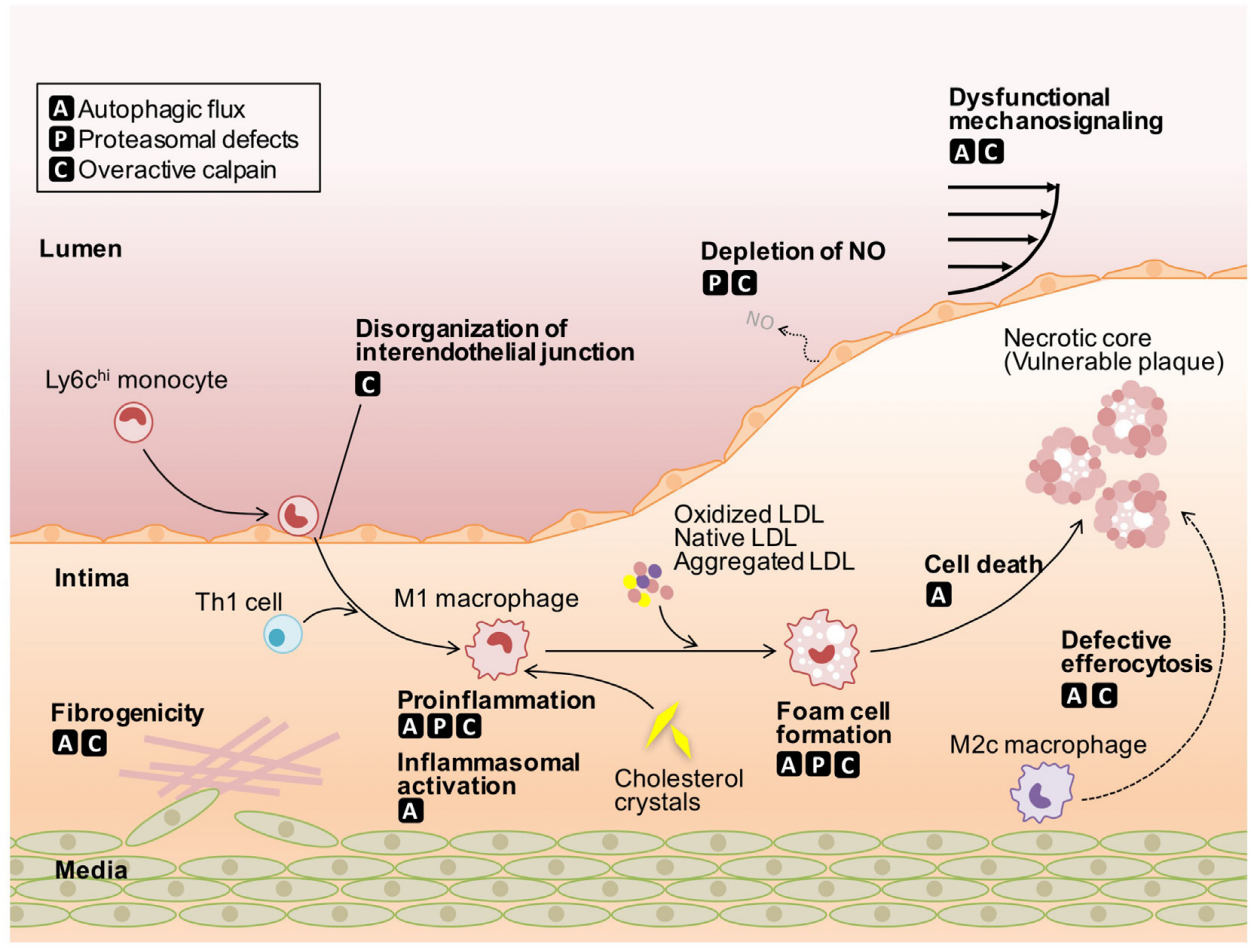
Roles of defective protein catabolism in the pathogenesis of atherosclerosis. Autophagic flux leads to insufficient mechanosignaling in endothelial cells (ECs), impaired phenotypic conversion in vascular smooth muscle cells (VSMCs), proinflammatory actions in macrophages, and defective efferocytosis, thereby accelerating the onset of atherosclerosis. Proteasomal defects induce the depletion of nitric oxide in ECs and harmful proinflammatory responses in macrophages, despite protecting cells from apoptotic and non-apoptotic cell death. Overactivation of conventional calpains leads to impaired EC integrity, VSMC phenotypic conversion, and increased macrophage atherogenicity. Calpain-6 contributes to efferocytic pathways in macrophages. LDL, low-density lipoprotein; NO, nitric oxide.

During the development of atherosclerosis, the dysfunction of ECs and fibrogenicity of VSMCs, as well as cholesterol deposition and proinflammatory responses in macrophages are driven by defective protein catabolism (Figure 1; Table 1). The cell type-specific contribution of these defects to atherosclerosis is discussed below.

**Table 1:** Protein catabolism defects and atherosclerosis-related events.

	Cell type	Events	Related processes	Reference
Autophagic flux	ECs	NO depletion	Mechanotransduction	(56)
		Endothelial NO synthase uncoupling	Mechanotransduction	(57)
	VSMCs	Fibrogenic response	VSMC transformation to synthetic phenotype	(64)
	Macrophages	Vascular inflammation	Inflammasome secretion of IL-1β	(17)
		Impaired efferocytosis	Efferocytic clearance of dead cells	(18)

Proteasomal defects	ECs	Impaired vasodilation	NO production	(25)
	Macrophages	Proinflammation	NF-κB activation (inhibitor κB degradation)	(76)
		Vascular protection?	Anti-apoptosis	(10)

Overactive calpain	ECs	Barrier dysfunction	VE-cadherin disorganization	(34)
		Endothelial disintegrity	NO depletion	(52)
	VSMCs	Arterial fibrosis	Upregulation of NF-κB signals	(65)
		Age-associated vascular fibrosis	VSMC transformation to synthetic phenotype	(66)
		Vascular calcification	VSMC transformation to synthetic phenotype	(67)
		Carotid restenosis	VSMC transformation to synthetic phenotype	(68)
	Macrophages	Vascular inflammation	Upregulation of NF-κB signals	(36)
		Impaired efferocytosis	mRNA splicing defects (calpain-6)	(37)

*ECs, vascular endothelial cells; VSMCs, vascular smooth muscle cells; NO, nitric oxide; IL-1β, interleukin-1β; NF-κB, nuclear factor-κB; VE-cadherin, vascular endothelial-cadherin*.

### Vascular ECs

Endothelial cells cover the luminal surface of blood vessels and act as a physical barrier to limit the infiltration of active plasma factors through the vascular wall. It is well known that the endothelial barrier is disorganized in atheroprone arteries because of inflammatory insults (1), which accelerate the recruitment of immune cells that subsequently promote proinflammatory responses in the vessels. It is noteworthy that immune cells can also infiltrate atheroprone vascular walls through the vasa vasorum (40). Previous studies identified three major types of interendothelial junctions: tight junctions, gap junctions, and adherence junctions (41). In human umbilical vein ECs, tight junctions are present in up to 20% of the total junctional complexes, while adherence junctions account for approximately 90% (42). Vascular endothelial (VE)-cadherin forms adherence junctions in a Ca^2+^-dependent homophilic manner (43). Furthermore, vascular permeability in mice was elevated by the administration of anti-VE-cadherin neutralizing antibody, whereas this treatment had no effect on other types of junctions in ECs (44); thus, adherence junctions have a pivotal role in barrier maintenance in ECs. Our previous investigations identified that administration of the calpain inhibitors calpeptin and *N*-acetyl-Leu-Leu-methional (calpain inhibitor II) recovered the dysfunction of the EC barrier in dyslipidemic mice (34). Mechanistically, a juxtamembrane domain of VE-cadherin is proteolyzed by calpain-2, thereby dissociating β-catenin from the VE-cadherin complex, leading to the destabilization of adherence junctions. As a result, macrophage infiltration and the subsequent development of atherosclerosis in the dyslipidemic mice were suppressed by pharmacological inhibition of conventional calpains. Calpain proteolyzes E-cadherin and N-cadherin; thus, calpain systems regulate the posttranslational processing of cadherin family proteins (45, 46). In contrast to the calpain family, the contribution of autophagy and proteasomal systems to atherosclerotic endothelial barrier dysfunctions has not been reported to date, although these pathways were reported to participate in the disorganization of tight junctions in septic pulmonary ECs (47) and ischemic cerebral ECs (48).

Endothelial nitric oxide (NO) production is crucial for ensuring vascular integrity. NO reacts with superoxide to neutralize its toxicity; therefore, a reduction of NO bioavailability facilitates ROS-dependent vascular inflammation. Furthermore, uncoupled endothelial nitric oxide synthase (eNOS), which generates superoxide radicals instead of NO, is largely responsible for ROS production even in atheroprone vessels (49, 50), in addition to xanthine oxidase and NADPH oxidase. It was reported that conventional calpains were responsible for physiological NO production in ECs. Indeed, calpain inhibitors *N*-acetyl-leucyl-leucyl-norleucinal (calpain inhibitor I) or calpeptin prevented vascular endothelial growth factor (VEGF)-driven NO production in cultured ECs (51). Furthermore, VEGF accelerated the translocation of calpain to the plasma membrane to form an ezrin-containing molecular complex. Accordingly, AKT, AMP-dependent kinase (AMPK), and eNOSs1179 were phosphorylated through the molecular complex, which is indispensable for NO production in ECs. In addition to VEGF-driven NO synthesis, disorders of NO production under pathophysiologic conditions might be caused by the dysregulation of calpain systems. Yu et al. documented that the administration of calpain inhibitor I increased the activity and protein expression of aortic eNOS in high fat diet-fed rats along with the elevation of NO (52) indicating excessive calpain activity may disturb NO synthesis in ECs. Herrmann et al. reported that in contrast to calpain inhibitors, the chronic administration of the proteasome inhibitor MLN-273 increased oxidative stress and exacerbated the development of atherosclerosis in pigs fed a high cholesterol diet (25). *In vitro* experiments further showed that inhibition of the proteasome prevented EC-dependent vasodilation in isolated coronary arteries. Collectively, protein catabolism pathways are critical for endothelial NO production. It appears that proteasomal systems ensure NO synthesis, while calpain systems act as a negative regulator for NO production in atheroprone vessels.

It is widely known that fluid shear stress, the blood flow-generated dragging force tangential to the EC surface, is a critical determinant for atherosclerosis susceptibility in blood vessels. This is because shear stress-dependent pathways in ECs are involved in barrier regulation and NO production (53). Interestingly, aortic arch, coronary artery, and aortic bifurcation, where blood flow is disturbed, are proatherogenic; thus, atherosclerosis susceptibility in blood vessels is largely dependent upon the vascular architecture because of the limited EC mechanotransduction in these regions (54). Yang et al. reported that physiological fluid shear stress sustained substantial autophagic activity in cultured ECs, but was reduced in cells subjected to low shear stress (55). Shear stress-induced eNOS phosphorylation and NO production were markedly blunted by autophagic deficiency (56). Similar to impaired NO production, the loss of autophagic activity potentiates ROS and cytokine production in ECs, suggesting that autophagy may play a suppressive role in eNOS uncoupling. Consistent with these results, autophagic inhibition by 3-methyladenine downregulated eNOS expression in sheared carotid arteries in rabbit, while autophagic activation by rapamycin potentiated eNOS expression (57). In addition to shear stress-dependent autophagic regulation, we previously showed that endothelial calpain-2 was activated in response to physiologic shear stress in cultured ECs (58). This activity was mediated through Ca^2+^ influx and phosphatidylinositol 3-kinase and was responsible for EC alignment in the direction of the blood flow. Interestingly, calpain-2 is enriched in specific vascular regions, such as the lesser curvature of the aortic arch and origin of vascular branch, in normal physiological murine aortae, where the blood flow is structurally disturbed (59). Thus, the autophagic and calpain systems orchestrate endothelial mechanotransduction.

### Vascular Smooth Muscle Cells

It was reported that cardiovascular defects such as hypertension and atherosclerosis are strongly related to age-associated alterations in arterial structure and function (60, 61). The hallmark of such disorders is the stiffening of large arteries caused by ECM remodeling and vascular calcification and is triggered by the phenotypic conversion of contractile VSMCs to a synthetic phenotype. Mechanistically, synthetic VSMCs orchestrate elastin fragmentation as well as the synthesis and degradation of collagen through ROS-dependent inflammatory cascades. It was reported that RhoA, TGF-β, Notch, and integrin–matrix pathways modulated SMC differentiation (62) and that platelet-derived growth factor (PDGF) orchestrated the conversion of VSMCs to a contractile state along with autophagic activation (63). The pharmacological intervention of autophagy appears to inhibit the PDGF-induced phenotypic conversion in VSMCs, indicating the contribution of autophagic signals to the conversion. Interestingly, VSMC-specific deficiency of autophagy exacerbated high fat diet-induced atherosclerosis in *Apoe^−/−^* mice, which was accompanied by a thickening of the fibrous cap (64). Furthermore, the autophagic flux resulted in an increase in collagen content, TGF-β expression, and matrix metalloproteinase-9 in VSMCs. Thus, autophagy in VSMCs is protective in atherogenesis, but augments plaque vulnerability.

In addition to autophagy, calpain systems were reportedly associated with the phenotypic regulation in VSMCs. The transgenic overexpression of calpastatin antagonized arterial fibrosis and hypertrophy in mice infused with angiotensin II (65) by reduced MMP levels and the downregulation of NF-κB signals in arterial media. Similarly, the age-associated overactivation of calpain-1 in VSMCs participated in the upregulation of MMP2 and VSMC motility (66). Furthermore, osteopontin and osteonectin levels in VSMCs were reduced by the overexpression of calpain-1 in rats, inducing the calcification and fibrosis in the arteries of aged rats (67). Furthermore, VSMC proliferation and collagen synthesis were accelerated during ligation-induced carotid restenosis in mice, which was opposed by the transgenic overexpression of calpastatin (68). Therefore, calpain systems have a pivotal role in inflammation-related fibrogenic responses in VSMCs.

### Macrophages

Macrophages exert an essential role in the pathogenesis and progression of atherosclerosis (69). Bone marrow-derived circulating monocytes can be divided into two subsets, LY6C^hi^ and LY6C^low^, in mice (70–72). The increased number of circulating monocytes in hypercholesterolemic mice is responsible for the expansion of the LY6C^hi^ subset (70), which is a major source of recruited macrophages in atheromas. In the early stage of atherosclerosis, circulating monocytes adhere to luminal ECs and migrate into the intimal layer according to the gradient of cytokines/chemokines (1). Subsequently, monocytes are differentiated into classically (M1) and alternatively (M2) activated macrophage subsets. M1 subset is mainly associated with inflammatory responses in atherosclerotic lesions and is derived from LY6C^hi^ monocytes (70–72). The M2 subset can be further divided into at least 4 subgroups, M2a, M2b, M2c, and M2d, which participate in tissue repair and inflammatory resolution in some instances (73). Foam cells in atherosclerotic lesions are mostly derived from the M1 subset, which is mediated through receptor-dependent and receptor-independent uptake of LDL cholesterol. In contrast, M2c macrophages incorporate cholesterol by engulfing dead cells efferocytically (74). The balance of M1 and M2 polarization can be disrupted in the presence of inflammatory burden in atherosclerotic lesions at least in part by disturbed poly (ADP-ribose) polymerase- or Dll4/Notch-related signals (73). Because cytosolic-free cholesterol exhibits robust cytotoxicity, the excessive incorporation of cholesterol results in cell death in macrophages. Accordingly, the acceleration of cholesterol uptake in macrophages in atheroprone vascular walls increases the number of dying macrophages, thereby forming cholesterol-enriched vulnerable atherosclerotic lesions (75). In addition to their defective cholesterol handling, atheroprone macrophages play central roles in innate immunity, including toll-like receptor and NOD-like receptor-mediated inflammasome signaling, in lesions (69). Although the protein catabolism pathway critically influences both cholesterol handling and inflammatory cascades in cells, cholesterol handling is not discussed in this issue. Please refer to our previous review articles for information on proatherogenic cholesterol handling in macrophages by defective cholesterol catabolism (12).

It was reported that macrophage-specific deficiency of autophagy by the deletion of *Atg5* facilitated atherogenesis in *Apoe^−/−^* mice (17). Interestingly, *Atg5*-deficient macrophages had increased oxidative stress and enhanced inflammasome secretion of IL-1β. The inflammasome systems in *Atg5*-null macrophages were potentiated by the addition of cholesterol crystals to the culture systems. It is likely that the inflammasome secretion of IL-1β by *Atg5*-null macrophages in atheromas may be potentiated by cholesterol crystals, because they are enriched in atherosclerotic lesions in *Atg5-*deficient mice. Furthermore, necrotic cores and oxidative stress in lesions of *Ldlr^−/−^* mice were enhanced by the macrophage-specific deletion of *Atg5* (18). Autophagic activity in isolated macrophages was potentiated by proatherogenic stressors, such as 7-ketocholesterol or 1-(palmitoyl)-2-(5-keto-6-octene-dioyl) phosphatidylcholine. Such autophagic activity suppressed apoptotic cell death by the reduction of endoplasmic reticulum stress and oxidative stress by NADPH oxidase 2. Furthermore, atherogenicity in macrophages was also mediated by proteasomal defects. For example, Brand et al. reported that the pharmacological inhibition of proteasomes prevented the proteolytic degradation of inhibitor κBα, an endogenous inhibitor of NF-κB transcriptional systems, by oxidized LDL in monocytes (76). This indicates that proteasomal inhibitors possess anti-inflammatory effects in atherosclerosis. In contrast, it was reported that oxidized LDL-stimulated macrophages showed increased ubiquitination activity, which suppressed apoptosis (10). Similarly, the complex mode of cell death in VSMCs was abrogated by 7-ketocholesterol-indced proteasomal activity (11). Thus, proteasomal activity may be involved in cell survival under stressed conditions, while it participates in cytotoxic inflammatory burden. In addition to the autophagic and proteasomal flux, the participation of calpain systems in inflammatory cascades of macrophages was proposed. It appears that calpain can proteolytically degrade inhibitor κBα, thereby accelerating NF-κB signaling (77); thus, the overactivation of calpain systems potentiates inflammatory responses. The transduction of *CAST* prevented the production of inflammatory cytokines in peritoneal macrophages isolated from high fat diet-fed *Apoe^−/−^* mice (36). Collectively, macrophages can be susceptible to proatherogenic burden by the overactivation of calpain systems. Whether defective protein catabolism influences macrophage polarity is currently unclear and, therefore, should be studied in the future.

Vulnerable atherosclerotic lesions harbor vast number of inflammatory cells, a necrotic core, and a thin fibrous cap (78). In particular, expansion of the cholesterol-enriched necrotic core by the accumulation of dead cells weakens the physical stability of plaques. It was reported that macrophages engulf and remove cellular corpses in lesions through efferocytic activity, thereby reducing the necrotic core (78). Furthermore, the efferocytic actions of macrophages participate in the reprogramming of cells toward anti-inflammatory phenotypes, leading to the resolution of inflammation. Importantly, the clearance of dead cells by efferocytosis is limited in advanced atheromas, because “eat-me/find-me” signals in apoptotic cells and phagocytosis pathways in macrophages are prevented in the lesions (78). Thus, the coordination of integrated cell death signals in dying cells and phagocytic signals in phagocytes are necessary for efferocytosis. In the case of mammary gland involution in pregnancy, efferocytic activity was accelerated in mammary epithelial cells, resulting in remodeling of the tissues and the production of milk (79). Mammary epithelial-specific *Atg7* deficiency markedly reduced efferocytic activity, Rac1 activity, and MertK expression; thus, autophagic signaling is associated with efferocytosis-associated tissue remodeling. In the case of atherosclerosis, autophagic defects caused by *Atg5* deficiency reduced the efficiency of the efferocytic clearance of dead cells, leading to an expansion of the necrotic core (18). It is likely that *Atg5* deficiency in dying cells, but not phagocytic macrophages, prevented the efficiency of efferocytosis. Therefore, the autophagic disorder in dying cells or phagocytic macrophages induced defects in efferocytosis, although this needs to be elucidated in the future. While the participation of conventional calpains in efferocytosis has not been reported to date, calpain-6 seems to be involved in this event. Indeed, the efferocytic clearance of dead cells by cultured macrophages was modestly inhibited by *Capn6* deficiency (37). Accordingly, the deletion of *Capn6* resulted in a reduction of necrotic cores in atheromas in *Ldlr^−/−^* mice, which was accompanied by the upregulation of Rac1 protein in the cells. Rac1 prevention by a pharmacological inhibitor suppressed efferocytic activity in macrophages (80); therefore, it is interpreted that efferocytic activity in lesional macrophages was prevented by calpain-6, probably through Rac1 downregulation. Because conventional calpains are associated with cellular dynamic processes, such as migration (81) and trafficking of intracellular vesicles (82), it will be interesting to investigate the participation of these calpains in efferocytosis.

## Targeting Defective Protein Catabolism in Atherosclerosis

As stated above, autophagic disorders cause insufficient mechanosignaling in ECs, impaired phenotypic conversion in VSMCs, inflammatory action in macrophages, and defective efferocytosis, thereby increasing the susceptibility to atherosclerosis (Figure 1). Therefore, recovering autophagic activity may be a promising treatment for the atherosclerosis burden. One possible candidate for atherosclerosis intervention is mTOR, a robust autophagic suppressor. mTOR inhibitors normally exhibit proautophagic activity thereby suppressing atherosclerosis (83). For example, atherosclerosis in rabbits fed a high cholesterol diet (84), or in *Apoe^−/−^* (85) and *Ldlr^−/−^* mice (86) was markedly inhibited by oral administration of the mTOR inhibitor rapamycin. Clinical trials of several mTOR inhibitors for cancer treatment are currently underway (87). Based on our current knowledge, it is expected that the use of mTOR inhibitors will also be beneficial for treating cardiovascular disorders.

Proteasomal activation induces harmful proinflammatory responses in macrophages but protects cells from apoptotic and nonapoptotic cell death, whereas proteasomal inhibition leads to the depletion of EC-derived NO (Figure 1). Thus, based on animal experiments, it is likely that inhibiting proteasomes will have a dual action. Accordingly, the clinical applications of proteasomal inhibitors for treating atherosclerosis are currently unclear, even though atherosclerotic diseases can be reversed by moderate levels of proteasomal inhibition (27). In addition to these inhibitors, endogenous activators of proteasomes, PA28, PA200, and PA700, as well as chemical proteasome activators, are currently available (88). The beneficial effects of PA28 overexpression in a neuronal cell model of Huntington’s disease were reported (89). Restoring proteasomal activity by activators should be investigated in future atherosclerosis studies.

Both calpain-6 and conventional calpains are involved in atheroprone events in lesional macrophages. Whereas conventional calpains lead to impaired EC integrity, phenotypic conversion of VSMCs and increased macrophage atherogenicity, calpain-6 contributes to cholesterol uptake and efferocytic processes in phagocytic macrophages (Table 1). Importantly, the majority of proatherogenic events can be prevented by loss-of-function calpains (12, 30), indicating that targeting calpain systems is a promising approach for atherosclerosis therapy. Clinical trials of candidate compounds for the prevention of conventional calpains are currently underway, especially for neurodegenerative diseases (30). Based on our knowledge from basic research, the repositioning of these agents to the cardiovascular field may be highly beneficial. Isozyme selectivity of available calpain inhibitors, in particular, those regarding unconventional isozymes, are mostly unclear. Furthermore, it is expected that available inhibitors will not block calpain-6 because this molecule exhibits physiologic/pathophysiologic functions through its capacity to bind to other functional molecules, but not through its proteolytic activity. Evaluation of the isozyme selectivity of available inhibitors among unconventional calpains and the development of subtype selective inhibitors will be indispensable for calpain-targeted therapy in the future.

In conclusion, respective protein catabolism pathways, autophagy, proteasomes, and calpain systems, have unique properties under proatherogenic conditions. It appears that autophagy and proteasomes have fundamental roles in cell survival, while calpains appear to be dispensable. In contrast, autophagy and proteasomal systems are limited during the progression of atherosclerosis, while proatherogenic stressors activate calpains in some instances. It is noteworthy that apoptotic cell death was accelerated by the calpain-mediated proteolysis of Atg5 (90). Therefore, exploring the interrelations among the catabolism pathways in atherosclerosis is required for a better understanding of the pathogenic roles of these protein catabolism pathways.

## Author Contributions

TM conceived, designed, appraised the literature, and wrote the manuscript. AM reviewed and revised the manuscript.

## Conflict of Interest Statement

The authors declare that the research was conducted in the absence of any commercial or financial relationships that could be construed as a potential conflict of interest.
